# The toxic effects of anabolic steroids “nandrolone decanoate” on cardiac and skeletal muscles with the potential ameliorative effects of silymarin and fenugreek seeds extract in adult male albino rats

**DOI:** 10.1186/s40360-023-00658-x

**Published:** 2023-03-15

**Authors:** Dalia Abd Elwahab Hassan, Sherien S. Ghaleb, Amr reda Zaki, Ahmed Abdelmenem, Shimaa Nabil, Mostafa Abdallah Abdel Alim

**Affiliations:** 1 Department of Forensic Medicine and Clinical Toxicology, Faculty of Medicine, Beni-Suef University, Beni Suef, 62511 Egypt; 2 Department of Forensic Medicine and Clinical Toxicology, Faculty of Medicine, Cairo University, Cairo, 62514 Egypt; 3 Department of Pathology, Faculty of Veterinary Medicine, Beni-Suef University, Beni Suef, 62511 Egypt

**Keywords:** Nandrolone Decanoate, Anabolic steroids, Toxicity, Silymarin, Fenugreek seeds extract

## Abstract

**Background:**

Anabolic steroids (AS) are commonly abused by body builders and athletes aiming to increase their strength and muscle mass but unfortunately, the long-term use of AS may lead to serious side effects. Nandrolone Decanoate is one of the Class II anabolic androgenic steroids which quickly spread globally and used clinically and illicitly. Our research was directed to assess the toxic effects of anabolic steroids on cardiac and skeletal muscles in male albino rats and to evaluate the potential ameliorative effects of fenugreek seeds extract and silymarin.

**Methods:**

Our research was done on 120 male albino rats that were allocated into 6 groups; group I: Served as a control group, group II: Received the anabolic steroid Nandrolone Decanoate, group III: Received silymarin orally, group IV: Received fenugreek seeds extract orally, group (V): Received the anabolic steroid Nandrolone Decanoate and silymarin and group (VI): Received the anabolic steroid Nandrolone Decanoate and fenugreek seeds extract. By the end of the study, rats were sacrificed, and blood samples were collected for biochemical analysis and autopsy samples for histopathological examination.

**Results:**

The anabolic steroids toxic effects on rats showed a significant decrease in serum High Density Lipoprotein (HDL) level and increase in cholesterol, triglycerides, and Low-Density Lipoprotein (LDL) levels. There was a significant elevation in cardiac troponin I level. As regards to histopathological examination of the cardiac and skeletal muscles, the study showed marked degenerative changes and necrosis. Both silymarin and fenugreek seeds extract provided a protective effect on the biochemical and histopathological changes. The antioxidant effects of silymarin and fenugreek seeds extract were evaluated on the heart, skeletal muscles and showed that, the tissue levels of Superoxide dismutase (SOD), Catalase and reduced glutathione (GSH) decreased in AS treated rats compared to the control group. On the other hand, the tissue Malondialdehyde (MDA) levels were elevated.

**Conclusions:**

Anabolic steroids have a toxic effect on the cardiac and skeletal muscles of albino rats with improvement by treatment with fenugreek seeds extract and silymarin.

## Background

Anabolic steroids can be used for many therapeutic purposes; however, their usage is usually related with several adverse effects. These side effects are generally associated with the dose as therapeutic doses appears to have little side effects while supra-physiological doses are associated with severe and serious side effects [1].

Nandrolone Decanoate is one of the Class II anabolic androgenic steroids which quickly spread globally and used clinically and illicitly and composed of 19-nortestosterone-derivates [2].

Nandrolone Decanoate is mainly metabolized by the enzyme 5α-reductase, into 5α-dihydronandrolone, 19-norandrosterone, and 19- noretiocholanolone. These metabolites can be detected in urine [3].

Nandrolone Decanoate is used clinically in burns, radiation therapy, surgery, trauma, and various forms of anemia [4].

Also, used for the treatment of chronic kidney disease, osteoporosis in postmenopausal women [5].

Nandrolone Decanoate is used in inoperable breast cancer, patients on long-term corticosteroid therapy, assistant to therapy for conditions characterized by a negative nitrogen balance [6].

Several studies were performed to evaluate the pharmacological and therapeutic benefits of fenugreek in treating different conditions like diabetes, dyslipidemia, indigestion and flatulence, inflammation, aging and cancer with variable results and mechanisms [7]**.**

Fenugreek also known as *Trigonella foenum-graecum* is a plant of Fabaceae family widely cultivated in Asia and Southern Europe. The whole plant is rich in protein, vitamins and minerals so used as a nutritious healthy vegetable. In folk medicine, it is used in treatment of various conditions like diabetes, fever, and epilepsy [8].

Also, silymarin extracted from *Silybum marianum* has been used as a healing plant in folk medicine against different medical conditions such as liver disorders, rheumatic diseases, kidney problems, gastrointestinal tract disorders, cardiac disorders, and fever [9].

Our research was directed to assess the toxic effects of AS on some biochemical, histopathological parameters of cardiac and skeletal muscles in male albino rats in addition to evaluation of the potential ameliorative effects of Silymarin and fenugreek seeds extract.

## Methods

The research was done according to the rules established by the Local Research ethical Committee for the care and use of laboratory animals present in Beni-Suef University on 120 male albino rats from the animal house of Al Nahda University, faculty of Pharmacy, Beni-Suef Governorate, Egypt.

### Experimental design

The weight of the rats ranged from 150–200 gm and were stabilized for 8 weeks in the animal house before the start of the experiments. Plastic cages were used for animals housing within a room of optimum temperature (22 ± 2 °C) and the humidity level were adjusted to be (50 ± 5%). All animals were exposed to a 12-h cycles of light and dark with free access to food and water.

Assessment of the animals’ weight was done then they were randomly allocated into one of six groups each contain 20 rats:

(I) Rats received corn oil orally, 1 mg/kg/day “Control group”.
(II) Rats received intramuscular (IM) Nandrolone Decanoate at a dose of 20 mg/kg/week.
(III) Rats received oral Silymarin 20 mg/kg/day.
(IV) Rats received the extract of fenugreek seeds orally 450 mg/kg/day orally.
(V) Rats received IM Nandrolone Decanoate and oral silymarin with the same previous doses.
(VI) Rats received IM Nandrolone Decanoate and the extract of fenugreek seeds orally with the same previous doses.

### Chemicals

The anabolic steroid used was in the form of Nandrolone decanoate (Nandurabolin 50 mg/ml from El Nile Pharmaceutical company. Each ampule is 1 ml oily solution prepared for intramuscular injection.

The dose used was 20 mg/kg/week for 8 weeks. According to the animal weight, the dose was adjusted every week [10].

Silymarin used was in the form of oral suspension (Hepaticum 50 mg/5 ml) from Medical Union Pharma Company (MUP).

The silymarin dose used was 20 mg/kg/day for 8 weeks. According to the animals’ weight, the dose was adjusted every week [11].

The crude of fenugreek seeds extract was done according to Sakr and Shalaby, 2014 [12]**.** Semi dried seeds obtained from a local market were washed by distilled water then dried at 40 °C in an oven. The seeds were then ground by the lab grinder and passed through a sieve to obtain the raw material powder. Aqueous extract was obtained by heating the powder for 5 min after soaking in drinking water followed by filtration. The freshly prepared extract was used orally with a dose 450 mg/kg/day [13].

At the end of the experiment all rats were sacrificed via decapitation with light ether anesthesia inhalation.

### Biochemical assessment

Blood samples were then collected for estimation of cardiac enzymes (cardiac troponin I) and Lipid profile (Total cholesterol, HDL, LDL, and triglycerides).

The tissues were used for assessment of the antioxidant capacity by measuring Catalase, reduced glutathione (GSH), (SOD) and (MDA) in cardiac and skeletal muscles.

Malonaldehyde (MDA) concentration was calculated calorimetrically according to [14] Uchiyama and Mihara (1978). Also, reduced glutathione (GSH) concentration was estimated calorimetrically using Ellman's reagent according to [15] Sedlak and Lindsay (1968). Further, superoxide dismutase (SOD) concentration was measured using [16] Paoletti et al. (1986).

Phosphate buffered saline solution at pH 7.4 used to perfuse the tissues. A blood clots and RBCs were removed from tissues using 0.16 mg/ml heparin added to the solution. For each gram of tissues, 5–10 ml cold buffer solution was used for tissue homogenization. The cold buffer solution was formed from1 mM EDTA, 1 mL/L Triton X-100 and 50 mM potassium phosphate at pH 7.4.

Following tissue homogenization, at 4000 rpm centrifugation was done for 15 min at 4 °C with the supernatant removed for analysis. The samples were preserved by freezing at -80 °C that made them stable for at least one month.

### Histopathological examination

For histopathological assessment, Autopsy samples were dissected and removed from the rats in all groups. Heart and Skeletal muscles samples fixed by 10% neutral formalin then washed to be dehydrated with ascending grades of alcohol.

Samples were then cleared using Xylen to be embedded in hard paraffin and serially sectioned with 5–6 μ thickness mounted on albumenized slides.

Before staining, the slides were kept at 37 °C to dry for 24 h. Hematoxylin and Eosin (H&E) stains used then examination by the light microscope was done.

### Statistical analysis

Analysis of data was performed using SPSS v. 25 (Statistical Package for Social science) for Windows.

Description of variables was presented as follows:

- Description of quantitative variables was in the form of mean, standard deviation (SD) for normally distributed variables.
- All variables were explored for normality and showed that they were normally distributed.
- One-way ANOVA test was used to detect the difference between the four groups regarding the scale variables and Tukey post hoc high significant degree was conducted for multiple comparisons between groups.
- The significance of the results was assessed in the form of P-value that was differentiated into:
  - Non-significant when *P*-value > 0.05.
  - Significant when *P*-value ≤ 0.05.

## Results

### Cardiac troponin I level in the studied groups (Table 1)

**Table 1:** Comparison between the studied groups regarding the serum troponin level

Serum Troponin I	Group I	Group II	Group III	Group IV	Group V	Group VI
Mean ± SD	0.8 ± 0.1^a^	5.5 ± 0.5^b^	0.8 ± 0.1^a^	0.5 ± 0.1^a^	0.9 ± 0.1^a^	0.7 ± 0.1^a^

*Means within the same row in carrying different litters are significant at (P* < *0.001) using Duncan’s multiple range tests. Each group contain 20 rats*

As shown in Table 1, There was a significant difference between group II and other groups (*P*-value < 0.001). The level of troponin I was increased in group II with mean ± SD (5.5 ± 0.5) while decreased in group IV with mean ± SD (0.5 ± 0.1).

### Lipid profile in the studied groups (Table 2)

**Table 2:** Comparison between the studied groups regarding the lipid profile level

Mean ± SD	Group I	Group II	Group III	Group IV	Group V	Group VI
Serum cholesterol	76.6 ± 4.1^a^	103.3 ± 3.9^b^	87.3 ± 3.45^c^	73.4 ± 3.4^a^	91.4 ± 3.6^d^	81.8 ± 2.1^a^
Serum triglycerides	78.7 ± 3.9^a^	94 ± 2.6^b^	81.7 ± 1.5^a^	72.5 ± 2.6^c^	81.9 ± 2.7^a^	77.9 ± 2.3^a^
Serum LDL	36.3 ± 1.2^a^	46 ± 2^b^	37.9 ± 1.1^a^	30.6 ± 1.8^c^	38.5 ± 1.5^a^	37.1 ± 1.2^a^
Serum HDL	48.3 ± 1.1^a^	40.6 ± 1.6^b^	52.3 ± 1.8^c^	56.5 ± 1.9^d^	52.1 ± 2.7^e^	54.4 ± 1.8^f^

*Means within the same row in carrying different litters are significant at (P* < *0.001) using Duncan’s multiple range tests. Each group contain 20 rats*

*LDL*: Low density lipoprotein

*HDL*: High density lipoprotein

As shown in Table 2, There was a statistically significant differences between the six groups regarding the serum cholesterol, triglycerides, HDL, and LDL levels (*P*-value < 0.001).

The level of serum total cholesterol was increased in group II with mean ± SD (103.3 ± 3.9) while decreased in group IV with mean ± SD (73.4 ± 3.4).

The level of serum triglycerides was increased in group II with mean ± SD (94 ± 2.6) while dcreased in group IV with mean ± SD (72.5 ± 2.6).

The level of serum LDL increased in group II with mean ± SD (46 ± 2) while decreased in group IV with mean ± SD (30.6 ± 1.8).

The level of serum HDL increased in group IV with mean ± SD (56.5 ± 1.9) while the decreased in group II with mean ± SD (40.6 ± 1.6).

### Changes in the oxidative stress and antioxidant marker of the heart in the studied groups (Table 3)

**Table 3:** Comparison between the studied groups regarding the heart antioxidants level

Mean ± SD	Group I	Group II	Group III	Group IV	Group V	Group VI
SOD	2.35 ± 0.11^a^	2.08 ± 0.06^b^	2.37 ± 0.16^a^	3.03 ± 0.17^c^	2.38 ± 0.08^a^	2.84 ± 0.15^d^
Catalase	1.66 ± 0.13^a^	1.27 ± 0.11^b^	1.68 ± 0.08^a^	1.69 ± 0.09^a^	1.65 ± 0.08^a^	1.62 ± 0.09^a^
MDA	2.22 ± 0.09^a^	2.39 ± 0.14^a^	2.11 ± 0.07^a^	2.17 ± 0.10^a^	2.12 ± 0.13^a^	2.18 ± 0.09^a^
GSH	1.64 ± 0.06^a^	1.46 ± 0.14^a^	1.86 ± 0.10^b^	1.77 ± 0.12^a^	1.75 ± 0.06^a^	1.72 ± 0.05^a^

*Means within the same row in carrying different litters are significant at (P* < *0.001) using Duncan’s multiple range tests. Each group contain 20 rats*

*MDA* Malondialdehyde

*SOD* Superoxide dismutase

*GSH* Reduced glutathione

As shown in Table 3, There was a statistically significant differences between the six groups regarding the heart SOD, catalase, and reduced glutathione (GSH) levels (*P*-value < 0.001).

The level of SOD increased seen in group IV with mean ± SD (3.03 ± 0.17) while decreased was in group II with mean ± SD (2.08 ± 0.06).

The level of catalase increased seen in group IV with mean ± SD (1.69 ± 0.09) while decreased in group II with mean ± SD (1.27 ± 0.11).

The level of MDA increased in group II with mean ± SD (2.39 ± 0.14) while decreased in group III with mean ± SD (2.11 ± 0.07).

The level of GSH increased seen in group III with mean ± SD (1.86 ± 0.10) while decreased in group III with mean ± SD (1.46 ± 0.14).

### Changes in the oxidative stress and antioxidant marker of the skeletal muscles in the studied groups (Table 4)

**Table 4:** Comparison between the studied groups regarding the skeletal muscles antioxidants level

Mean ± SD	Group I	Group II	Group III	Group IV	Group V	Group VI
SOD	2.8 ± 0.1^a^	2.5 ± 0.1^a^	2.9 ± 0.1^a^	3.3 ± 0.2^b^	2.8 ± 0.7^a^	3.1 ± 0.2^c^
Catalase	1.2 ± 0.1^a^	1 ± 0.1^b^	1.3 ± 0.1^a^	1.25 ± 0.1^a^	1.1 ± 0.03^a^	1.1 ± 0.1^a^
MDA	2.4 ± 0.1^a^	2.8 ± 0.2^b^	2.3 ± 0.1^a^	2.54 ± 0.1^a^	2.47 ± 0.1^a^	2.5 ± 0.2^a^
GSH	1.9 ± 0.05^a^	1.5 ± 0.1^b^	2 ± 0.2^a^	2.3 ± 0.1^c^	1.9 ± 0.1^a^	2.2 ± 0.2^a^

*Means within the same row in carrying different litters are significant at (P* < *0.001) using Duncan’s multiple range tests. Each group contain 20 rats*

*MDA* Malondialdehyde

*SOD* Superoxide dismutase

*GSH* Reduced glutathione

As shown in Table 4, There was a statistically significant differences between the six groups regarding the skeletal muscles SOD, catalase, MDA and GSH levels (*P*-value < 0.001).

The level of SOD increased seen in group IV with mean ± SD (3.3 ± 0.2) while decreased in group II with mean ± SD (2.5 ± 0.1).

The level of catalase increased in group III with mean ± SD (1.3 ± 0.1) while decreased in group II with mean ± SD (1 ± 0.1).

The level of MDA increased in group II with mean ± SD (2.8 ± 0.2) while decreased were in group III with mean ± SD (2.3 ± 0.1).

The level of GSH increased in group IV with mean ± SD (2.3 ± 0.1) while decreased in group II with mean ± SD (1.5 ± 0.1).

### Histopathological changes in the heart of the studied groups (Fig. 1)

**Fig. 1:**
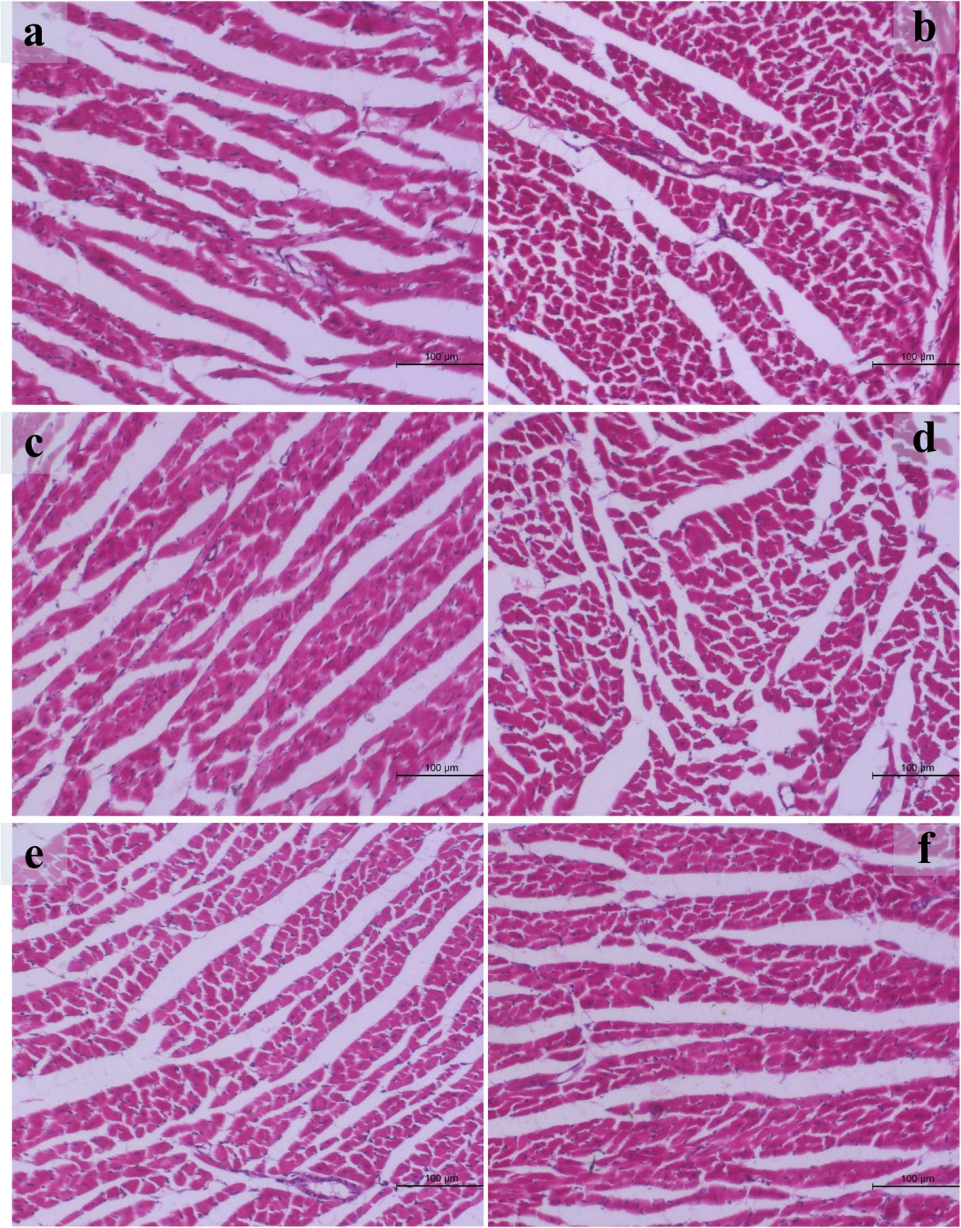
Histopathological changes of cardiac muscle in different groups. **a** Section in the heart of group I showing normal histological structure (H&E X-100). **b** Section in the heart of group II showing severe degenerative changes and necrosis (H&E X-100). **c** Section in the heart of group III showing normal histological structure (H&E X-100). **d** Section in the heart of group IV showing normal histological structure (H&E X-100). **e** Section in the heart of group V showing moderate degenerative changes and hyalinosis (H&E X-100). **f** Section in the heart of group VI showing mild degenerative changes (H&E X-100)

Detailed cardiac pathological lesions were showed in (Fig. 1). The cardiac lesions were degenerative changes and necrosis of the cardiac muscles (hyalinosis).

In group I, III and IV a normal histological structure of cardiac muscles could be found appearing as long cylindrical striated cells with centrally located one or two large oval nuclei (Fig. 1a), (Figs. 1c) and (Fig. 1d) respectively.

Severe degenerative changes and necrosis of cardiac muscles could be found in group II (Fig. 1b).

Group V showed moderate degenerative changes and hyalinosis of cardiac muscles (Fig. 1e), while group VI was associated with mild degenerative changes of the cardiac muscles in comparison to the other groups (Fig. 1f).

### Histopathological changes in the skeletal muscles of the studied groups (Fig. 2)

**Fig. 2:**
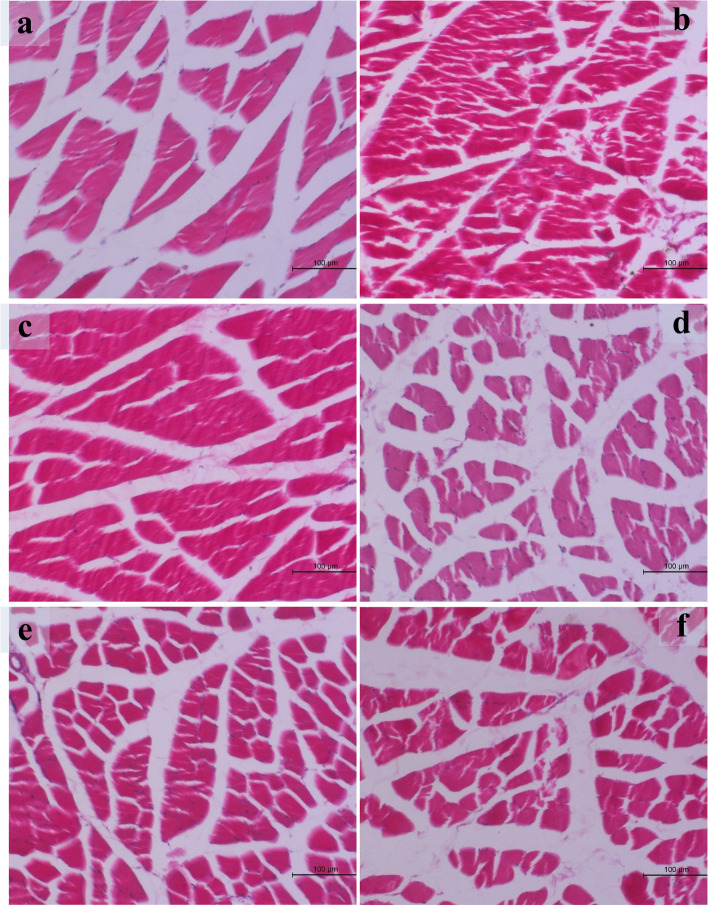
Histopathological changes of skeletal muscle in different groups. **a** Section in the muscle of group I showing normal histological structure (H&E X-100). **b** Section in the muscle of group II showing severe degenerative changes and nuclear pyknosis of the sarcoplasm (H&E X-100). **c** Section in the muscle of group III showing normal histological structure (H&E X-100). **d** Section in the muscle of group IV showing normal histological structure (H&E X-100). **e** Section in the muscle of group V showing quite improvement of the degenerative changes (H&E X-100). **f** Section in the muscle of group VI showing quite improvement of the degenerative changes (H&E X-100)

Detailed muscular lesions are showed in (Fig. 2). Histopathological examination of the skeletal muscle obtained from group I, III and IV were normal (Fig. 2a), (Fig. 2c) and (Fig. 2d) respectively.

Group II exhibited severe pathological lesions (severe degenerative changes with nuclear pyknosis of the sarcoplasm) (Fig. 2b).

On the other hand, quite improvement of degenerative changes of the musculature mainly in group V and VI. (Fig. 2e) (Fig. 2f) respectively.

## Discussion

The use of AS showed a dramatic increase in the recent years especially by young adults aiming to increase their power, their body mass and weight and to have a better appearance improving their self-esteem. Also, AS have been used for decades by athletes and body builders for the same purpose. On the other hand, many side effects were reported with their illicit abuse [17].

Our work was performed to assess the toxic effects of AS on the heart and the skeletal muscles and assessment of the ameliorative effects of silymarin and fenugreek seeds extract.

As shown in Table 1, There was a significant difference between group II and other groups. Highest level of troponin I was seen in group II (5.5 ± 0.5) while the lowest level was in group IV (0.5 ± 0.1).

The AS treated rats showed a significant elevation in troponin levels. Also, there were non-significant decrease in Silymarin and fenugreek seeds extract. When, silymarin and fenugreek seeds extract were given with AS the levels of serum troponin decreased with no significant differences.Similarly, Kulaksiz and Sefa, 2019 [18] reported an increase in the serum levels of troponin in testosterone treated rats.

Cardiac troponin I (cTnI) is a cardiac enzyme which is released when ether is a damage in the myocytes and considered a sensitive factor of these damages. The levels of this enzyme showed no significant changes in research done on mice treated with silymarin alone [19].

Silymarin could keep the membrane integrity, limiting the leakage of enzymes revealing the cardio-protective effect [20].

The activity of cardiac enzyme biomarkers including troponin showed a significant decrease with fenugreek seeds by 27% indicating the cardio-protective effect of fenugreek seeds [21].

Kamble and Bodhankar, 2014 [22] explained that the cardio-protective effect of fenugreek seed could be due to trigonelline, saponins, 4 hydroxyisoleucine presence, and high fiber contents. Also, may be due to fenugreek scavenging properties of the free radical. These properties are attributed to the active hydrogen-donating ability of the hydroxyl substitutions [12].

As regards to the lipid profile, rats received AS showed a significant elevation in serum cholesterol, triglycerides and LDL levels and a significant decrease in serum HDL levels. Silymarin treated rats showed a significant increase in serum cholesterol and HDL levels. Also, there was elevation in serum triglycerides and LDL levels with no significant differences. In rate treated with fenugreek seeds extract, there was a significant decrease in the serum triglycerides and LDL levels. Also, the serum cholesterol levels decreased but with no significant differences. On the other hand, serum HDL levels increased with significant difference.

Like the results of this study was that reported by Silva et al., 2018 [23] who demonstrated that AS could significantly raise the serum triglycerides, cholesterol, and LDL levels. Unlike, their effect on HDL that showed a significant reduction in serum level.

The abuse of AS in supra-physiological doses is strongly associated with abnormal levels of plasma lipoproteins showing a decreased level of HDL and increased levels of LDL and cholesterol levels [24].

Treatment with fenugreek seeds extract showed improvement in lipid profiles, including decrease in serum triglycerides, total cholesterol, and LDL levels. Also, there was a significant elevation in HDL levels [25]. This effect could be because of sapogenins agent that play a role in decreasing the synthesis of cholesterol and fatty acids. Also, they increase the excretion of cholesterol in bile lowering the serum cholesterol levels [26].

Also, Roberts, 2011 [27] demonstrated that saponins could decrease the cholesterol levels by inhibiting the absorption of cholesterol and enhance its excretion in bile by forming large particles with bile salts. The excess soluble fibers in fenugreek extract may lead to delay in the absorption of fat and carbohydrate adding to the hypolipidemic effect. Also, the mannose presents in the extract reduce the synthesis of cholesterol.

Metwally et al., 2009 [28] reported increase in serum cholesterol, LDL, triglycerides, and HDL levels regarding the effect of silymarin on lipid profile.

Gobalakrishnan et al., 2016 [29] explained the different mechanisms for silymarin induced hypo-cholesterolemic effect. Among these effects are the increased the production of LDL receptors on hepatic cells increasing the clearance of plasma LDL. Also, silymarin increases the conversion of cholesterol into bile acid. However, silymarin has no effect on the absorption of cholesterol.

The antioxidant effects of silymarin and fenugreek seeds extract were evaluated on the heart, skeletal muscles and showed that, the tissue levels of SOD, Catalase and reduced glutathione decreased in AS treated rats compared to the control group. On the other hand, the tissue MDA levels were elevated.

Similarly, previous studies [30] and [31] reported that an increase of the lipid peroxidation and decrease of the enzymatic activity of glutathione reductase and SOD of different tissues in adult male rats because of exogenous testosterone intake.

Frankenfeld et al., 2014 [32] showed a decrease in the activity of catalase enzyme after AS (nandrolone decanoate) intake.

High doses of nandrolone decanoate are metabolized by cytochrome P450 mono-oxygenases. This results in production of reactive oxygen species leading to the upregulation followed by exhaustion of the antioxidants enzymes activity [33].

Furthermore, anabolic steroids enhance the activity of lipase enzyme which in turn increases the rate of lipolysis [34]. which in turn increases the availability of long chain fatty acids for mitochondrial oxidation and production of ATP leading to development of the lipid peroxidation and generation of ROS [35].

As regards to silymarin, our results were in consistence with the study performed by Avci et al., 2017 [36] about the effect of silymarin on MDA and SOD levels in the heart of rats. They demonstrated an increase in the activity of SOD and decrease in MDA levels denoting a protective effect against lipid peroxidation and oxidative damage. Similarly, Aktas and Ozgocmen, 2020 [37] revealed in their study an increase in the activity of glutathione enzyme and decrease in MDA levels in cardiac tissue of rats treated by silymarin.

Surai, 2015 [38] reported that silymarin is a potent antioxidant inhibiting the lipid peroxidation and prevents the reduction of glutathione enhancing the activity of antioxidants enzymes.

This effect could be clarified by its effect on maintaining the integrity of cell membranes against the oxidative damage of ROS [39].

This antioxidant properties probably due to the presence of silydianin, silybin, silychristin and flavolignans which are chemical bioactive compounds present in silymarin [40].

Regarding fenugreek seeds extract, Bafadam et al., 2021 [41] revealed an increase in the activity of SOD and catalase in heart tissue of rats and decrease in lipid peroxidation augmented by the decreased MDA levels in hearts tissues of rats treated with germinated fenugreek seed. Also, Arshadi et al., 2015 [42] showed an increase in the activity of cardiac antioxidant enzymes like reduced glutathione, catalase, and SOD.

Fenugreek seeds extract antioxidant properties are mainly because of the presence of the biologically active compounds flavonoids and polyphenols. Its major active antioxidant compounds are isovitexin and flavones of vitexin [43].

Histopathological examination of cardiac muscle in our study showed that, the most observed lesions were degenerative changes and cardiac muscles necrosis (hyalinosis) which was severe in rats treated with AS. On the other hand, normal histological structure of cardiac muscles could be found in rats of the control group and those received silymarin and fenugreek seeds extract. Improvement of the toxic effects induced by AS was observed with treatment of silymarin and fenugreek seeds extract with mild degenerative changes were seen with treatment by fenugreek seeds extract and moderate degenerative changes with treatment by silymarin.

Histopathological examination of the skeletal muscle in control group rats and rats treated with silymarin and fenugreek seeds extract were normal. Severe degenerative changes associated with nuclear pyknosis of the sarcoplasm were seen in rats treated with nandrolone decanoate. The toxic effect of AS could be improved with treatment by silymarin and fenugreek seeds extract. Also, Kahal and Allem, 2018 [44] showed in their study elongation, severe degeneration and may be rupture of the cardiac muscle. Also, these results coincide with Hassan et al. (2009) [45]“AS sustaining induces severe ischemic necrosis and degeneration of the cardiac muscle fibers in male albino rats.”

Abdelhafez, 2014 [46] showed highly degenerated muscle fibers with areas of hemorrhage and widened endomysium. Also, she demonstrated a numerous pyknotic and karyolytic nuclei.

Also, Elgendy et al.,2018 [10] reported hypertrophy and degeneration of both cardiac and skeletal muscles and explained this by its effect on the androgen receptors that are widely distributed in different types of muscles.

The defensive effect of silymarin against cardiac damage was studied by Avci et al.,2017 [36] who reported that silymarin improve the cardiotoxic histopathological effects induced by cyclophosphamide. Other study performed by Aktas and Ozgocmen, 2020 [37] showed that silymarin preserves the histological structure of the heart and effectively promotes the antioxidant defense system against valproic acid induced injuries.

Also, El-Shitany et al., 2008 [47] demonstrated an improvement in the histopathological effects in Adriamycin induced cardiotoxicity when combined with silymarin and explained this by the antioxidant activity of silymarin inhibiting the lipid peroxidation and promoting antioxidant enzymes activity. Mendoza et al., 2020 [48] demonstrated a protective effect of silymarin on cardiac and skeletal muscle injuries due to its potent antioxidant effects.

## Conclusions

Anabolic Steroids have a toxic effect on cardiac and skeletal muscles associated with alteration of the biochemical markers, oxidative stress, and histopathological changes. However, fenugreek seeds extract and silymarin improved the toxic effects of AS on the cardiac and skeletal muscles with better results of fenugreek seeds extract.

## Data Availability

All data generated or analysed during this study included in this published article.

## Abbreviations

AS: Anabolic Steroids
MDA: Malondialdehyde
SOD: Superoxide dismutase
GSH: Reduced glutathione
IM: Intramuscular
LDL: Low density lipoprotein
HDL: High density lipoprotein
